# Inhaled Carbon Monoxide Protects against the Development of Shock and Mitochondrial Injury following Hemorrhage and Resuscitation

**DOI:** 10.1371/journal.pone.0135032

**Published:** 2015-09-14

**Authors:** Hernando Gomez, Benjamin Kautza, Daniel Escobar, Ibrahim Nassour, Jason Luciano, Ana Maria Botero, Lisa Gordon, Silvia Martinez, Andre Holder, Olufunmilayo Ogundele, Patricia Loughran, Matthew R. Rosengart, Michael Pinsky, Sruti Shiva, Brian S. Zuckerbraun

**Affiliations:** 1 Department of Critical Care Medicine, University of Pittsburgh, Pittsburgh, PA, United States of America; 2 The Center for Critical Care Nephrology^,^ University of Pittsburgh, Pittsburgh, PA, United States of America; 3 Department of Surgery, University of Pittsburgh, Pittsburgh, PA, United States of America; 4 Department of Pharmacology, University of Pittsburgh, Pittsburgh, PA, United States of America; 5 Vascular Medicine Institute, University of Pittsburgh, Pittsburgh, PA, United States of America; 6 VA Pittsburgh Healthcare System, Pittsburgh, PA, United States of America; Georgia Regents University, UNITED STATES

## Abstract

**Aims:**

Currently, there is no effective resuscitative adjunct to fluid and blood products to limit tissue injury for traumatic hemorrhagic shock. The objective of this study was to investigate the role of inhaled carbon monoxide (CO) to limit inflammation and tissue injury, and specifically mitochondrial damage, in experimental models of hemorrhage and resuscitation.

**Results:**

Inhaled CO (250 ppm for 30 minutes) protected against mortality in severe murine hemorrhagic shock and resuscitation (HS/R) (20% vs. 80%; P<0.01). Additionally, CO limited the development of shock as determined by arterial blood pH (7.25±0.06 vs. 7.05±0.05; P<0.05), lactate levels (7.2±5.1 vs 13.3±6.0; P<0.05), and base deficit (13±3.0 vs 24±3.1; P<0.05). A dose response of CO (25–500 ppm) demonstrated protection against HS/R lung and liver injury as determined by MPO activity and serum ALT, respectively. CO limited HS/R-induced increases in serum tumor necrosis factor-α and interleukin-6 levels as determined by ELISA (P<0.05 for doses of 100–500ppm). Furthermore, inhaled CO limited HS/R induced oxidative stress as determined by hepatic oxidized glutathione:reduced glutathione levels and lipid peroxidation. In porcine HS/R, CO did not influence hemodynamics. However, CO limited HS/R-induced skeletal muscle and platelet mitochondrial injury as determined by respiratory control ratio (muscle) and ATP-linked respiration and mitochondrial reserve capacity (platelets).

**Conclusion:**

These preclinical studies suggest that inhaled CO can be a protective therapy in HS/R; however, further clinical studies are warranted.

## Introduction

Trauma represents one of the leading causes of injury, disability and mortality worldwide. Death from traumatic injury is often attributable to the hemodynamic, inflammatory and metabolic consequences of hemorrhagic shock[1], and the cellular and organic response to injury[2]. Accordingly, the understanding of these processes is fundamental to further the development of targeted interventions aimed at reducing cell and tissue injury, limiting organ dysfunction and ultimately reducing mortality.

One of the most highly conserved adaptive mechanisms to cellular injury, is orchestrated by the heme oxygenase-1 (HO-1) pathway[3]. HO-1 is an enzyme that catalyzes the degradation of heme into carbon monoxide (CO), biliverdin, and iron. In the setting of shock, up regulation of HO-1 has been shown to minimize inflammation and to restore normal adaptive mechanisms[4–6]. In agreement with this, others and we have shown that induction of HO-1 limits cellular damage and organ dysfunction, whereas inhibition results in exacerbation of injury[7, 8]. Importantly, CO seems to be at the forefront of these protective effects[9]. Others and we have previously shown that administration of inhaled low dose CO to mice subjected to hemorrhagic shock, not only protected from organ injury and modulated the inflammatory response, but also paradoxically reduced tissue hypoxia[10–12].

The purpose of the present study is to test the hypothesis that inhaled CO protects against organ injury and inflammation in different models of hemorrhagic shock and in different species, and furthermore, that such protection is associated with CO-induced limitation of oxidative injury and reduction in mitochondrial injury. In addition, these studies intend to provide pre-clinical evidence that administration of inhaled CO is safe from the hemodynamic and hematologic standpoint.

## Materials and Methods

### Ethics statement

All experiments were approved by the Institutional Animal Care and Use Committee of the University of Pittsburgh, protocol numbers (13061614 and 13092382).

### Murine model of hemorrhagic shock

Anesthesia, surgical preparation and resuscitation protocol. Male C57BL/6 mice (23–27g) were anesthetized with pentobarbital (70 mg/kg and kept at a constant temperature using a heating pad set at 37°C throughout the experiment. Under sterile conditions, both femoral arteries were dissected and cannulated. One catheter was connected to a high frequency blood pressure analyzer for animal monitoring and data gathering. The other was connected to a motorized, two-way peristaltic pump for bleeding and shed blood re-infusion. A stabilization period were baseline heart rate, pulse pressure, systolic and diastolic pressures, and MAP values were collected over a 15-min period. Bleeding was performed using a modification of the Wiggers method[13]. “Moderate shock” was achieved by bleeding to a targeted MAP of 25 mmHg, hemorrhaged over a 15-min period to achieve these pressures. Blood pressure was kept at this level for 120 minutes. MAP was sustained by re-infusion and withdrawal of shed blood during the experiment as indicated. Animals were resuscitated with Lactated Ringers at two times the volume of maximal shed blood after 120 minutes. Animals were euthanized 4 hours after resuscitation, and serum and tissues were obtained for analysis. Sham mice were anesthetized and cannulated in a similar fashion. The ‘Moderate shock’ model was used for measurements of organ injury (ALT, Myeloperoxidase activity, and lipid peroxidation), as well as systemic inflammation by serum cytokines. N = 8 mice per experimental group. In “Severe shock”: mice were bled to a MAP of 20 mmHg and maintained at this pressure for 30 minutes. For determination of measurements of shock by arterial blood gas (n = 8 mice per group), mice were sacrificed at the end of this thirty minute time point. For determination of compensation endpoint (n = 10 mice per group), mice were maintained at a MAP of 20 until the time point that they would no longer maintain this pressure without re-infusion of volume. This time point defines the “compensation endpoint[14].” Mice were sacrificed once the compensation endpoint was determined.

For survival experiments the hemorrhage protocol was modified to achieve a higher mortality rate (n = 20 mice per group). Briefly, mice were hemorrhaged to a MAP of 20 mmHg (“severe shock”) and maintained at this pressure for 30 minutes. Mice were then resuscitated with shed blood to a MAP of 25 and maintained at this pressure for an additional hour. Following this mice were resuscitated with lactated Ringers at 2 times the maximal shed blood volume.

Mice were monitored during these experiments continuously during the surgical procedure then checked upon at least every two hours post-operatively. Thew were monitored for mobility, and signs of distress. Buprenex was administered every 6 hours for pain at the dose of 0.10 mg/kg. If a mouse appeared in distress or pre-morbid they were euthanized with CO_2_ asphyxiation.

Mouse models and experimental modesl are summarized in S1 Fig and S1 Table.

#### Intervention

Mice were randomized to receive no treatment or inhaled CO for 30 minutes, at different doses (i.e. 50, 100, 250, or 500 ppm premixed in 21% oxygen and 79% nitrogen). This was delivered via a flow through chamber (700 cubic centimeter volume) at a flow rate of 2 liters/minute. Carbon monoxide was initiated 90 minutes into the (120 minute) hypotensive period in the ‘moderate shock’ model. For experiments determining pH, lactate, base deficit, and compensation endpoint, CO treatment (250 ppm) was initiated once the MAP reached 20 mmHg and then maintained for 30 minutes. For survival experiments, mice were randomized to receive room air or inhaled CO (250 ppm) for the last 30 minutes of the (90 minute) hypotensive period.

### Porcine Model of Hemorrhagic Shock

#### Anesthesia and Surgical preparation

The pigs were sedated using TKX (Telazol, Ketamine, Xylazine) at 1 ml/50lbs intramuscular injection in the thigh and transported to the surgery suite. The pigs then received oxygen and isofluorane 2–4%, and underwent endotracheal intubation. Swine were ventilated on volume control mode, with tidal volume of 8–10ml/kg, inspired fraction of O_2_ of 0.4 and were sustained under anesthesia with isofluorane 2–2.5%. The animals were monitored with EKG monitor and pulse-oximeter in the tail, and maintenance fluids (5% dextrose and normal saline) were started through a peripheral ear vein at 1ml/kg/hr. After cleaning the surgical areas with iodopovidone, the swine were then instrumented with a right internal jugular pulmonary artery catheter through an 8 French introducer, a right femoral vein 10 French introducer and a right femoral artery triple lumen catheter. An incision was then made in the left groin to expose the rectus femoris muscle and the vastus medialis muscle to collect muscle biopsies throughout the experiment. This area was covered with wet gauze to avoid desiccation. Animals were then allowed to stabilize for 30 minutes before starting hemorrhage.

#### Hemorrhagic shock and resuscitation

After the 30-minute stabilization period, the swine were bled at a rate of 20 ml/min via femoral arterial catheter until the MAP decreased to 30 mmHg. Bleeding was re-initiated at 60ml/min if the animal was able to compensate to a MAP of 40 mmHg, and was continued until the 30 mmHg target was reached. This step was repeated whenever compensation to 40 mmHg was achieved in order to maintain the animals between MAP of 30–40 mmHg for a maximum of 90 minutes. Total shed blood volume was determined throughout the duration of the experiment. Animals were resuscitated with a 1:1 volume of Hextend to shed blood volume either at 90 minutes or if they fulfilled decompensation criteria, defined as MAP below 30 mmHg for 10 minutes or below 20 mmHg for 10 seconds. Following initial volume infusion, further resuscitation with lactated ringer’s was dictated by an algorithm based on arterial pressure waveform contour analysis-derived Stroke Volume Variation (SVV) to assess volume responsiveness (FloTrac, Edwards Lifesciences, LLC, Irvine, CA, USA), and aiming at re-establishing baseline MAP. Volume responsiveness was defined as an SVV > 10%. The pigs were then observed for up to 4 hours (S2 Fig). Pigs were monitored throughout for pain or distress. Pigs were euthanized by increasing to 5% inhaled isoflurane for 5 minutes, followed by a central intravenous bolus of KCl 80 mEq.

#### Intervention

Swine were randomly allocated to either of two groups: Standard resuscitation or standard resuscitation (Shock group) and inhaled CO (CO group). Randomization was done in blocks of 10 animals. Of the 10 animals, one was allocated blindly to receiving sham surgery, which consisted of cannulation as previously described, without hemorrhage. Given that blinding was not possible, allocation to either group was disclosed to the investigators performing the study only after 55 minutes into the hypotensive period in order to avoid any bias during the surgical or hemorrhage phases. Animals allocated to CO received a pre-mixed gas containing 250 parts per million (ppm) in 40% oxygen after 60 minutes into the shock period delivered through the anesthesia circuit.

#### Myeloperoxidase (MPO) activity

MPO activity in the lungs in the murine moderate shock model was determined four hours after resuscitation as described by Anderson et al[15]. Lungs were excised, washed in saline, and frozen in liquid nitrogen. Samples were thawed and homogenized in 20 mM/L potassium phosphate (pH 7.4). Samples were centrifuged at 15,000*g* for 30 min at 4°C. The pellet was then resuspended in 50 mM/L potassium phosphate (pH 6.0) containing 0.5% hesadecyltrimethylammonium bromide. Samples were sonicated and then centrifuged 15,000*g* for 10 min at 4°C. Supernatant (5 mircoliters) was then added to 196 microliters of reaction buffer containing 530 nM/L *O*-dianisidine and 150 nM/L H_2_O_2_ in 50 mM/L potassium phosphate (pH 6.0). Light absorbance at 490 and 620 nm was read and compared with standards. Protein content in the samples was determined by bicinchoninic acid assay. Results were corrected per microgram of protein.

#### Murine serum cytokine, ALT measurements, and arterial blood gas measurements

Serum levels of the cytokines IL-6 and TNF-alpha were measured in the murine moderate shock model four hours after resuscitation using enzyme-linked immunoabsorbant assay (ELISA; R&D Systems, Minneapolis, MN) according to the manufacturer’s instructions. Alanine aminotransferase (ALT) was determined using an iSTAT Analyzer (Abbott, Princeton, NJ). Arterial blood gases were obtained from the femoral artery and analyzed using an iSTAT Analyzer and blood gas cartridges from Heska (Loveland, CO).

#### Glutathione assay

Reduced glutathione (GSH) and the oxidized disulfide dimer (GSSG) were measured in liver of mice from the moderate shock model four hours after resuscitation using the Glutathione assay kit from Cayman Chemical (Ann Arbor, MI) as per the manufacturers instructions and adjusted for protein concentration.

#### Cell culture

Primary hepatocytes were isolated and purified from C57BL/6 mice (Jackson, Bar Harbor, Maine) and cultured as described previously[16]. Highly purified hepatocytes (>98% purity and >98% viability by trypan blue exclusion) were suspended in Williams medium E supplemented with 10% calf serum, 1 μM insulin, 2 mM l-glutamine, 15 mM HEPES (pH 7.4), 100 U/mL penicillin, and 100 microgram/mL streptomycin. The cells were plated on collagen-coated tissue culture plates at a density of 2 × 10^5^ cells/well in 12-well plates for cell viability analysis or 5 × 10^6^ cells/100-mm dish for Western blot and enzyme assays. After 18 h of preculture, the cells were further cultured with fresh medium containing 5% calf serum and used for experiment. All cell culture experiments were repeated in at least triplicate in cells harvested from separate cell isolations. Experiments that were performed under hypoxic (1% O_2_) conditions were carried out within a humidified anaerobic chamber (Coy Laboratory Products, Grass Lakes, MI). The chamber was digitally set at the required O_2_ concentration and was maintained at 37°C by a thermostatic controller.

#### Hepatocyte hypoxia determination

Primary mouse hepatocytes were seeded onto collagen cross-linked coverslips. For *in vitro* measurement of hepatocyte hypoxia, the nitroimidazole hypoxyprobe-1 was used as per the manufacturer's instructions, and mean fluorescence was determined in 10 high-powered fields per coverslip (n = 5 coverslips from 4 independent experiments per group). At the end of specified treatments, cells were rinsed 3 times with cold PBS, and then fixed in 2% paraformaldehyde in PBS for 1 hour.

#### Hepatic lipid peroxidation

Liver tissue from the murine moderate shock model four hours after resuscitation was fixed in 2% paraformaldehyde for 2 hours, dehydrated in 30% sucrose for 12 hours, and then frozen were sectioned (7 microns) onto gelatin-coated slides. Tissue was then blocked in 5% non-immune goat serum in PBS for 30 minutes at room temperature. Primary antibody to 4-hydroxy-2-nonenal (4-HNE, Abcam) was added to sections for 1 hour at room temperature. After washing, sections were incubated with fluorescent secondary antibodies for one hour (1:1000, Jackson ImmunoResearch Laboratories). Slides were then coverslipped using Gelvatol (23 g poly[vinyl alcohol 2000], 50 ml glycerol, 0.1% sodium azide to 100 ml PBS). Sections were imaged on a Fluoview 1000 confocal scanning microscope (Olympus, Melville,NY). Imaging conditions were maintained at identical settings within each antibody-labeling experiment with original gating performed using the negative control.

#### Measurements of respiration

Mitochondrial function in pigs and mice from striated muscle. In porcine hemorrhagic shock this was assessed in biopsies of striated muscle tissue obtained from the left rectus femoris muscle taken at the end of the stabilization period (baseline), 60 minutes into shock, immediately after resuscitation, and at the end of observation. Samples were processed immediately after collection. In mice, respiration was determined form muscle biopsies in the left leg prior to experimentation and then from the same muscle groups of the right thigh, 4 hours after resuscitation in the moderate shock model. Oxygen consumption in the muscle biopsies were immediately measured using a Clark-type oxygen electrode (Instech Laboratories, Plymouth Meeting, PA) in the presence of succinate (for State 4 measurements) and ADP (for State 3 measurement). Respiratory control ratio (RCR) was calculated as State 3/State 4.

Platelet or hepatocyte respiration or mitochondrial function was determined by placing cells in XF24 cell culture plates (Seahorse Biosciences, MA, USA) in a final volume of 250 microliters. Briefly, platelets from porcine shock experiments as described above were isolated from whole blood at the end of stabilization (baseline) and at the end of the observation period. Oxygen consumption rates were measured at each time point at basal rates, and after the addition of oligomycin (1 micrograms/ml), or FCCP (1micromoles) as previously described (Cardenes, Blood 2014). Alternatively, primary mouse hepatocytes exposed to normoxia or hypoxia (1% oxygen) for 30 minutes with or without concurrent CO treatment (250 ppm). Oxygen consumption rates were then determined.

#### Statistical Analysis

Results are expressed as mean±standard error of the mean (SEM). SigmaPlot (Systat Software, Inc., Point Richmond, CA) was used for the statistical analysis using either Students T-test for pairwise comparisons of two groups or one-way analysis of variance for significance and Tukey’s post hoc test when performing pairwise comparison with multiple treatment groups. Statistical significance comparing survival was determined using the Kaplan-Meier method. Significance was established at P<0.05.

## Results

**Fig 1:**
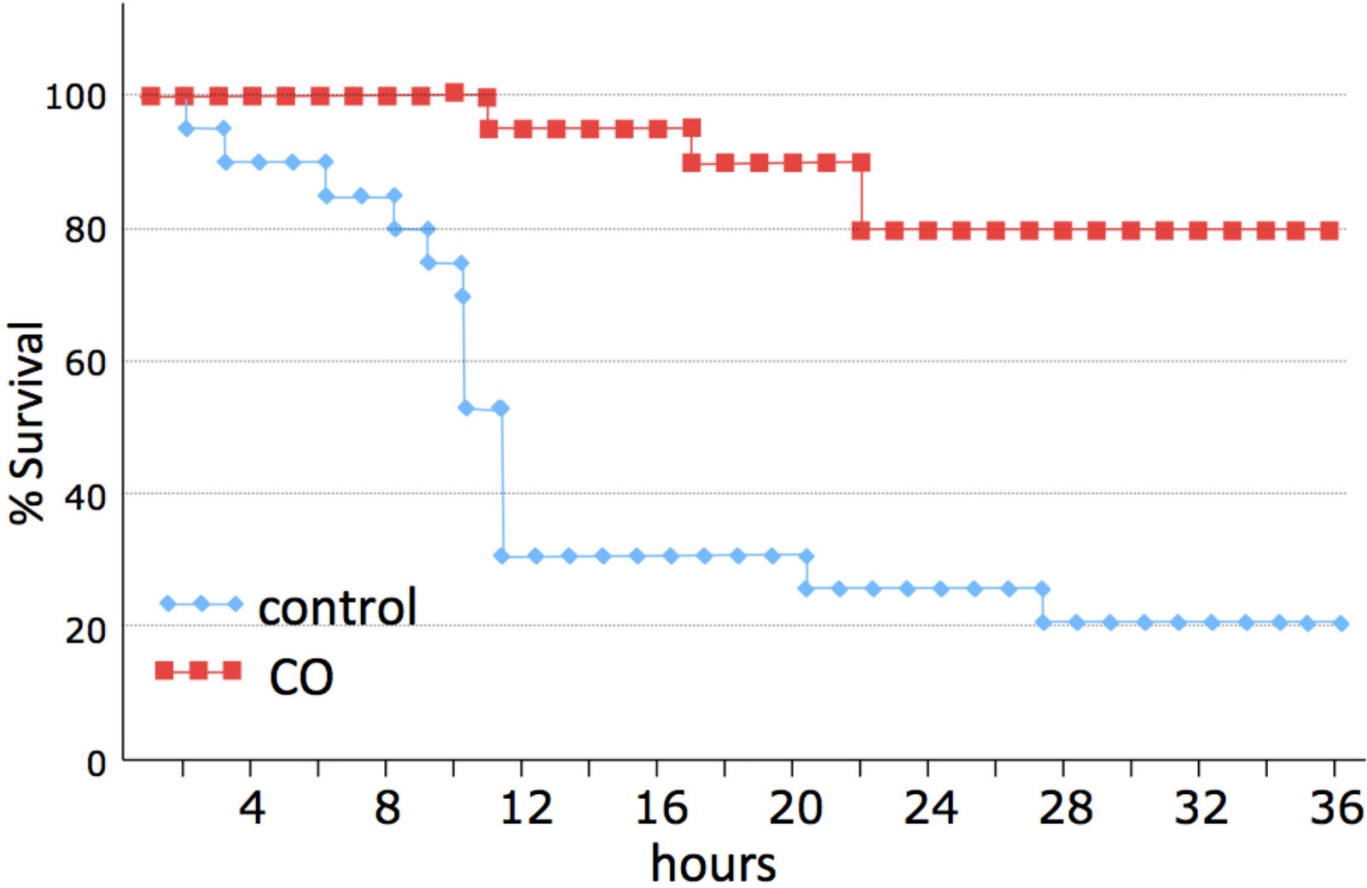
CO therapy protects against mortality in a model of murine hemorrhagic shock and resuscitation. A severe hemorrhagic shock and resuscitation model resulted in 80% mortality by 36 hours. CO therapy (250 ppm) limited mortality to 20% (P<0.05). Kaplan-Meier method was used to compare survival.

Inhaled carbon monoxide protected against death in a severe model of murine hemorrhagic shock and resuscitation. Survival was determined in a murine model of severe HS/R. The mouse model was modified as described above to achieve a relatively high mortality within 12 hours. Survival was determined over 36 hours as shown in **Fig 1**. No animals died during surgical procedures. Eighty percent of mice treated with standard resuscitation (shock group) died as compared to 20% of mice in the CO group (P<0.01 compared to shock alone; n = 20 mice). The highest mortality in the Shock group was seen early as 70% had died by 12 hours.

Carbon monoxide prevented hemorrhage-induced shock and delayed hemodynamic decompensation is a murine severe shock model. In order to determine the influence of hemorrhage with or without CO on the development of shock, mice were hemorrhaged to a MAP of 20 and treated with or without CO (250 ppm for 30 minutes). Hemorrhage resulted in signs of shock as measured by arterial pH, base deficit, and lactate (P<0.05) in both groups, however, treatment with CO limited these hemorrhage-induced changes as seen in **Table 1**. Furthermore, treatment with CO delayed reaching the compensation endpoint (defined as the time when mice bled to a pressure of 20 mmHg would require the re-infusion of volume to maintain this blood pressure) as compared to mice not receiving CO (52±7 minutes vs. 39±5 minutes, P<0.05; n = 10 mice per group). These data suggest that CO protects against the development of oxygen debt and can delay cardiovascular collapse.

**Table 1:** Arterial blood gas measurements in mice bled to and maintained at a MAP of 20 mm Hg for 30 minutes with and without inhaled CO (250 ppm). Sham mice underwent anesthesia and surgical manipulation without hemorrhage. Mice were bled to a pressure of 20 mm Hg over 15 minutes. CO therapy was started once a pressure of 20 mm Hg was reached.

	Sham	Sham + CO	Shock	Shock + CO
pH	7.27±0.05	7.29±0.04	7.05±0.05*	7.25±0.06^§^
Base deficit	5.4±2.7	6.1±3.6	24±3.1*	13±3.0^§^
Lactate	4±1.3	4.2±2.1	13.3±6.0*	7.2±5.1^§^

*P<0.05 compared to sham mice.

^§^P<0.05 compared to shock mice.

**Fig 2:**
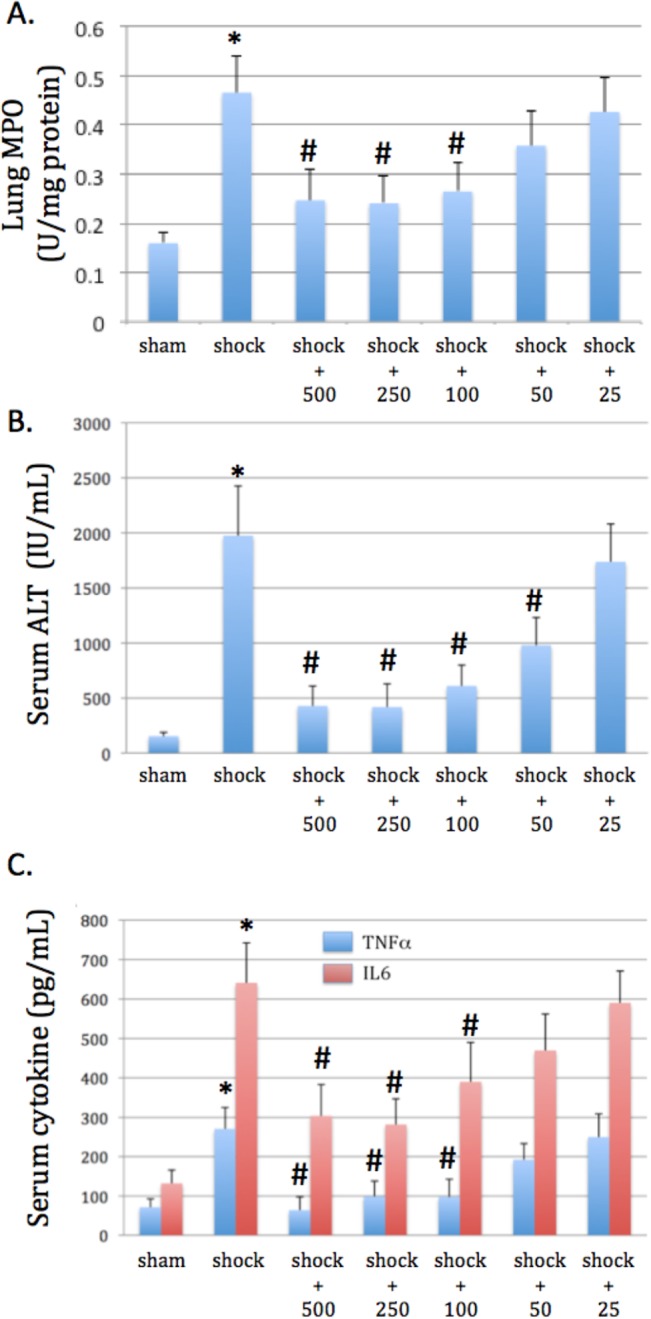
CO protects against organ injury and inflammation in a dose dependent fashion in murine model of hemorrhagic shock and resuscitation. Lung myeloperoxidase activity (MPO; **A.**) and serum ALT (**B.**) at 4 hours after resuscitation in mice demonstrates lung and liver injury, respectively. CO limits this injury in a dose-dependent fashion when treated for 30 minutes (25–500 ppm) starting 90 minutes into hypotension. C. Serum TNF-alpha and IL-6 levels were also increased by hemorrhagic shock and resuscitation at a 4 hour time point, and CO therapy limited these markers of inflammation in a dose dependent fashion. Results are mean±SEM for 8 mice per group. *P<0.05 compared to sham and #P<0.05 compared to shock. ANOVA was utilized for above comparisons.

Inhaled carbon monoxide protected against tissue injury in murine model of moderate hemorrhagic shock and resuscitation. We have previously demonstrated that inhaled CO as adjunct of standard resuscitation protected against organ injury in the setting of hemorrhagic shock[10]. In the present study we investigated whether escalating doses of CO would result in incremental organ protection in a dose-response fashion in the murine moderate shock model. Organ damage was assessed by serum ALT for liver dysfunction, lung myeloperoxidase activity as a marker of pulmonary neutrophil infiltration, and serum Interleukin 6 (IL-6) and Tumor Necrosis Factor-alpha (TNFa) levels as surrogates of global inflammation, all determined four hours after resuscitation. Escalating doses of CO from 50 to 500 ppm significantly prevented shock induced liver injury, pulmonary neutrophil infiltration and limited global inflammation (**Fig 2A–2C**, respectively). Finally, doses below 100 ppm for this duration of treatment were ineffective at providing any type of protection. These data are important from a clinical translational standpoint, as concentration and time lag before initiation of treatment provide insight into future potential uses in the field and pre-hospital settings.

**Fig 3:**
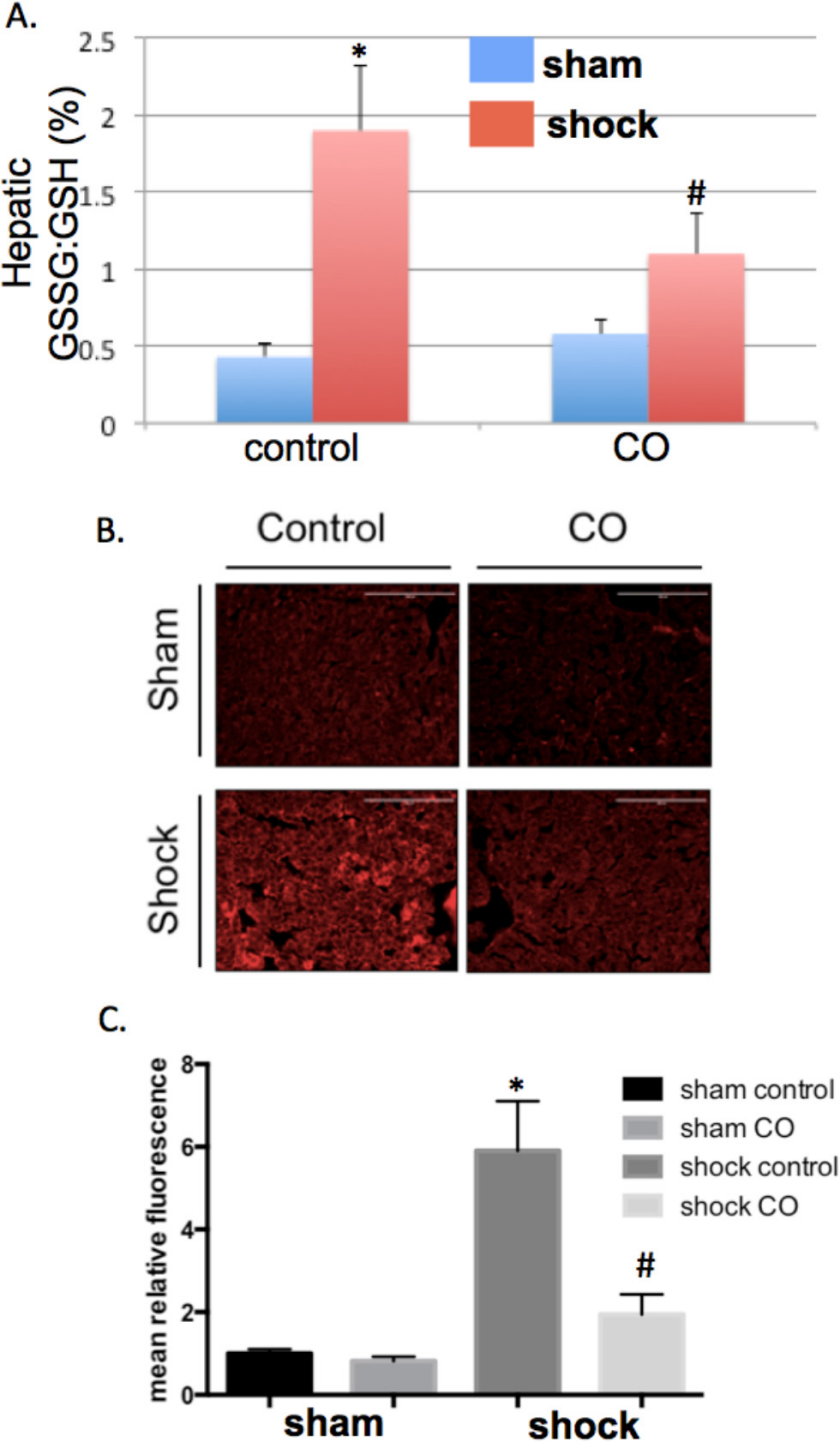
Hemorrhagic shock and resuscitation-induced oxidant stress is limited by CO. **A.** The ratio of oxidized glutathione (GSSG) to reduced glutathione (GSH) is increased in the liver of mice following HS/R (0.43±0.08 vs. 1.9±0.42; *P<0.05 compared to sham mice). CO treatment limited this increase in GSSG:GSH (1.1±0.27; #P<0.05 compared to shock mice). Results are mean±SEM for 8 mice per group. **B, C.** Representative immunohistochemistry and quantification of mean fluorescence of lipid peroxidation in liver tissue staining using 4-HNE at 4 hours after resuscitation in sham and shock mice with or without CO. ANOVA was utilized for above comparisons.

In addition to the above, we explored the effects of CO in protecting organs from HS/R-induced oxidative injury. This was examined in the liver tissue four hours after resuscitation by assessing the extent of lipid peroxidation and by measuring oxidized glutathione (GSSG) to reduced glutathione (GSH) ratios. HS/R resulted in oxidative injury and increased GSSG:GSH in hepatic tissue, as well as increased lipid peroxidation determined by 4-HNE staining (**Fig 3A–3C**). Importantly, CO at 250 ppm limited hepatic lipid peroxidation and oxidative injury.

**Fig 4:**
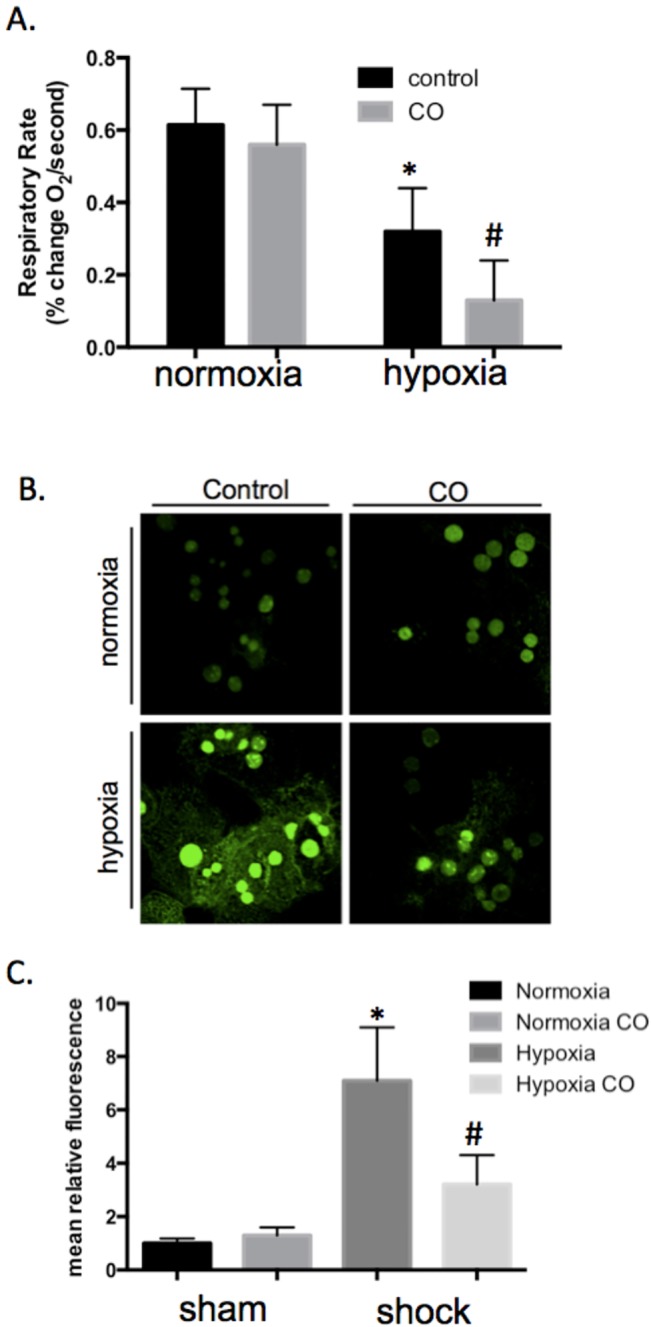
CO decreases oxygen consumption and limits the development of cellular hypoxia in hepatocytes in vitro. A. Oxygen consumption rates of primary murine hepatocytes were demonstrated *in vitro* in normoxic cells or in hepatocytes immediately following 30 minutes of hypoxia. CO treatment (250ppm) occurred during this normoxic or hypoxic periods. Hypoxia decreased oxygen consumption rates (*P<0.01 compared to normoxic cells) and this was further decreased by CO therapy (#P<0.05 compared to hypoxia alone). Results of four independent experiments, with each condition performed in triplicate. **B, C.** Representative immunocytochemistry and quantitative mean fluorescence of hypoxyprobe staining in hepatocytes under normoxic, normoxic+CO, hypoxic, or hypoxic +CO conditions for 30 minutes. Increased green staining represents increased cellular hypoxia. ANOVA was utilized for above comparisons.

CO decreased oxygen consumption in isolated hepatocytes in hypoxic conditions. The influence of CO on cellular oxygen consumption and availability in the setting of hypoxia was examined next. Cellular oxygen consumption rates were measured in primary mouse hepatocytes treated in normoxia and hypoxia (1% O_2_) with and without CO (250 ppm). Hypoxia induced a significant decrease in O_2_ consumption rate, which was further inhibited by the administration of CO as shown in **Fig 4A**. In addition, the influence of CO on relative intracellular oxygen levels was determined in primary hepatocytes by staining for the formation of nitroimidazole-protein binding adducts using hypoxyprobe staining. As **Fig 4B and 4C**demonstrate, the drop in intracellular O_2_ levels during hypoxia (increased fluorescence), was ameliorated by treatment with CO, suggesting that CO diminished hypoxia-induce decreases in intracellular oxygen. This may be secondary to changes in oxygen consumption as described above.

**Fig 5:**
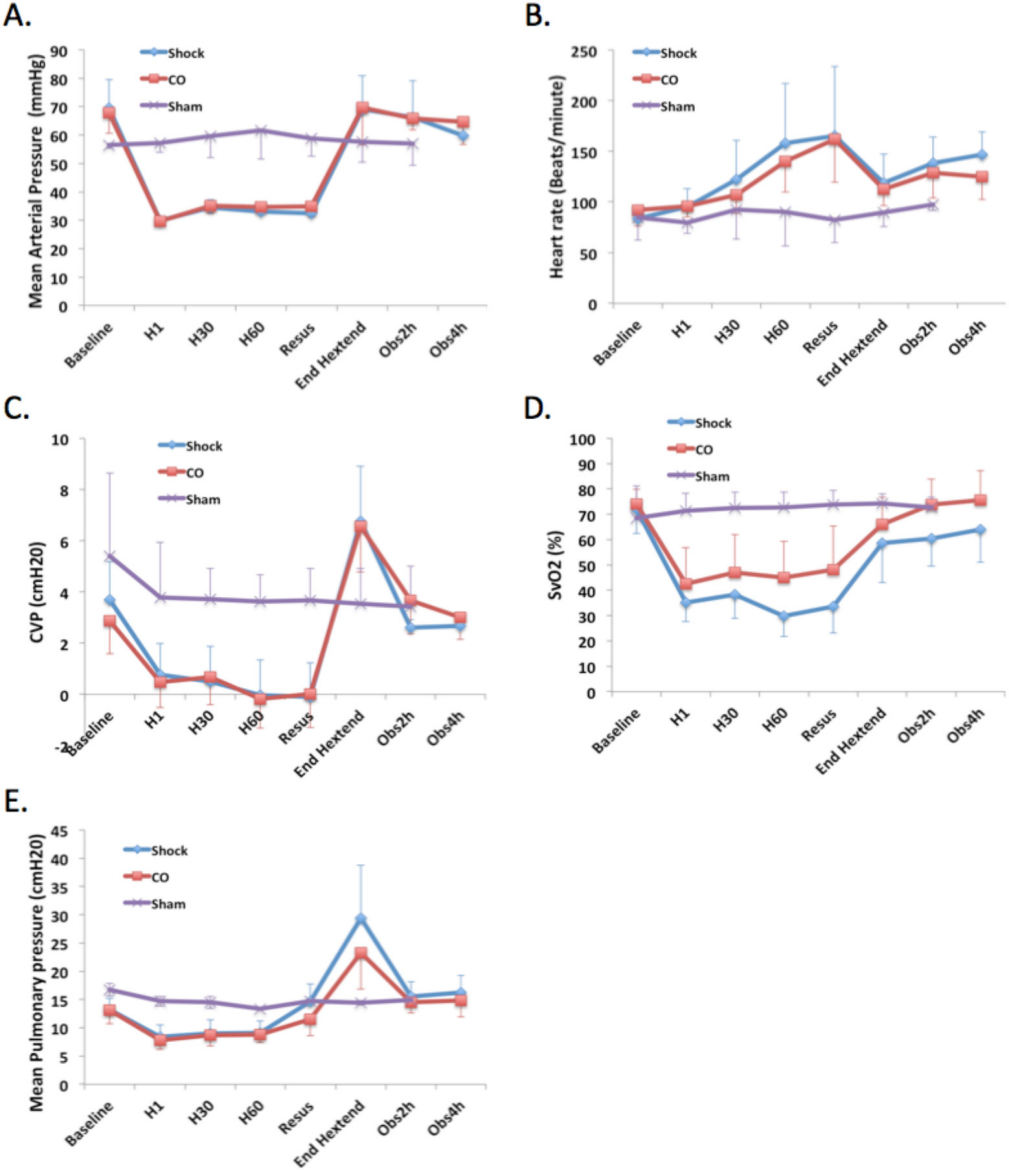
CO has minimal influence on gross cardiovascular parameters in porcine hemorrhagic shock and resuscitation. Hemodynamic data are shown at time points throughout the experiments [baseline, end of hemorrhage (H1), 30 minutes into hypotension (H30), 60 minutes into hypotension (H60), immediately prior to resuscitation (resusc), at the end of the initial hextend bolus (hextend), 2 hours into the resuscitation (Obs2h), and 4 hours into the resuscitation (Obs4h)]. Data is shown for mean arterial pressure (MAP, **A**.), heart rate (**B**.), central venous pressure (CVP, **C.**), mixed venous saturation (S_V_O_2_%, **D**.), and mean pulmonary arterial pressure (**E**.). Expected changes in hemodynamics are seen in shock and resuscitation, with no significant influences demonstrated in the CO treated pigs. ANOVA was utilized for above comparisons.

Carbon monoxide did not influence macro-hemodynamic parameters in porcine hemorrhagic shock and resuscitation. A porcine model of hemorrhagic shock and resuscitation was utilized to determine comprehensive evaluation of hemodynamics, as well as repeated measurements on mitochondrial function over time in the same animal study subject. A concern with CO treatment is the potential to influence hemodynamics, in part due to potential vasodilation. In this model of porcine HS/R, the use of CO had no influence in MAP, cardiac output or oxygen-based macro-hemodynamic parameters (**Fig 5**). Neither did CO have any measurable effects on stroke volume variation (SVV), heart rate (HR), or mean pulmonary artery pressure (MPAP) when compared to animals in the shock group. The MAP target of 30 mmHg (Time point H0) was reached after a median of 33 minutes (range 23–55 minutes), with no time difference between groups (*P* = 0.20). Of note, three was no statistically significant difference in the final volume of blood withdrawn during the hypotensive period (S2 Table).

Although this study was not powered to detect differences in cardiovascular collapse rates and survival, animals receiving CO showed a trend towards improved outcome. Seven pigs in the shock group (n = 15) hemodynamically decompensated (MAP < 30 mmHg for 10 minutes or < 20 mmHg for 10 seconds) requiring resuscitation prior to the 90 minute time-point, vs. 3 animals in the CO group (n = 12) (p = 0.42). Similarly, 4 animals in the shock group died prior to completion of the observation period, vs. 1 pig in the CO group (p = 0.34) (S3 Fig).

Hemoglobin levels dropped as anticipated with hemorrhage and there was no influence of CO on the shed blood volume. As expected, CO treatment increased carboxyhemoglobin levels as compared to the shock group (4.54±0.58% vs. 2.65±0.23%). However, this increase was modest, and within safe limits.

**Fig 6:**
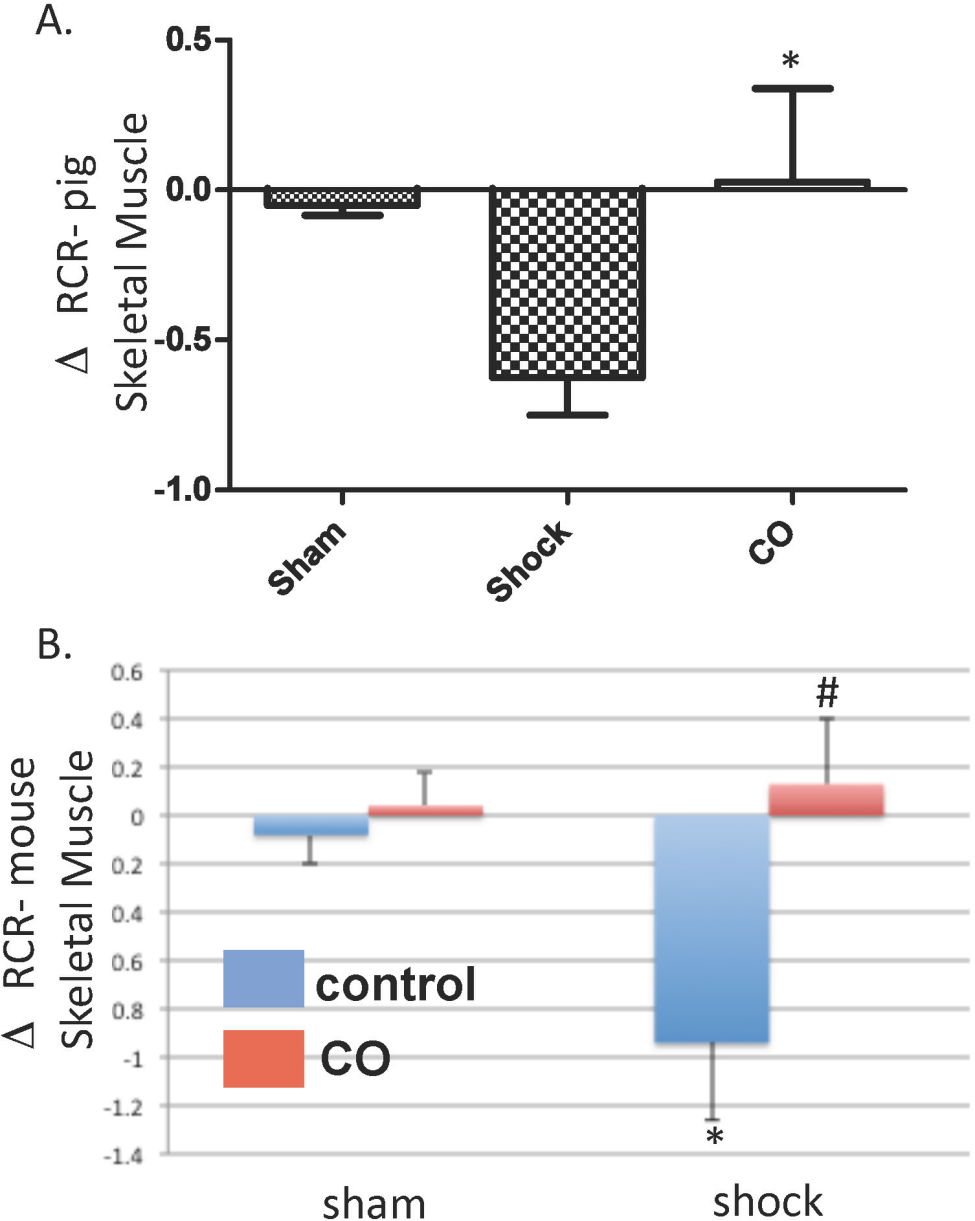
Hemorrhagic shock and resuscitation-induced skeletal muscle mitochondrial injury was limited by CO therapy. A. The change in respiratory control ratio (RCR; state 3:state4) from baseline to 2 hours after resuscitation in the porcine model demonstrated that HS/R led to mitochondrial injury. CO treatment resulted in an overall increase in the mean RCR, representing decreased mitochondrial injury (*P<0.05 compared to HS/R). B. Changes in RCR in murine thigh skeletal muscle from baseline to 2 hours after resuscitation demonstrated mitochondrial injury following HS/R, and inhaled CO protected against this injury (N = 8 mice per group; *P<0.05 compared to baseline; #P<0.05 compared to control treated-HS/R mice). ANOVA was utilized for above comparisons.

CO protected against tissue mitochondrial injury in hemorrhagic shock or hypoxia. Given that HS/R and the release of damage associated molecular patterns (DAMPs) during trauma can directly impair mitochondrial function[17–20], and that such impairment can result in cellular injury and organ dysfunction, the effect of HS/R and CO treatment on mitochondrial respiratory status in striated muscle and in platelets was determined. Respiratory control ratio (RCR), a global measure of the efficiency of mitochondrial oxidative phosphorylation, was determined in striated muscle at baseline, 60 minutes into shock, immediately after resuscitation and at the end of the observation period in the porcine model. During shock and immediately after resuscitation there was no evidence of functional mitochondrial injury in any group. However, at the end of observation RCR decreased in the shock group (-0.64±0.17) and was maintained in the CO-treated shock group (0.07±0.31; *P≤0.05 compared to shock) relative to baseline, suggesting that CO prevented mitochondrial uncoupling (**Fig 6A**). Similarly in murine hemorrhagic shock, RCR decreased from baseline at two hours following resuscitation (-0.94±0.32; *P≤0.05 compared to sham) and CO protected against these changes (#P≤0.05 compared to shock; **Fig 6B**).

**Fig 7:**
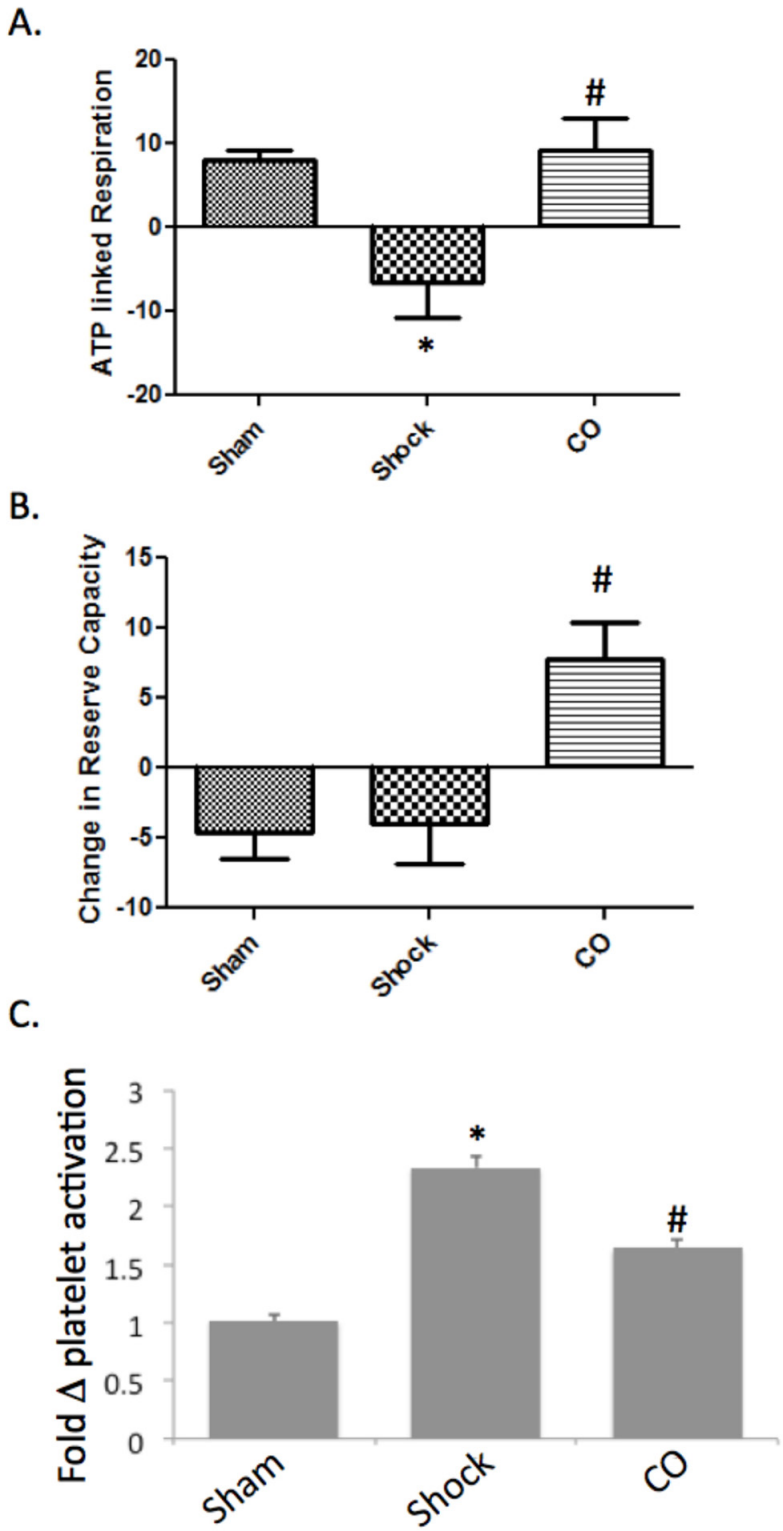
CO protects against hemorrhagic shock and resuscitation-induced platelet activation and mitochondrial injury. **A**. HS/R results in decreased ATP linked respiration (*P<0.05 compared to sham), while CO treatment prevented these changes (#P<0.05 compared to HS/R). **B**. HS/R had a minimal effect on mitochondrial reserve capacity, while CO treated HS/R pigs demonstrated an increase in this parameter (*P<0.05 compared to sham and HS/R) **C**. HS/R increased platelet activation by 2.33±0.1 fold over sham pigs at a 2 hour time point as determined by staining for CD62p by FACS (*P<0.05 compared to sham). CO treatment limited this activation to only a 1.64±0.08 increase over sham (#P<0.05 compared to HS/R). n = 7–11 pigs per group in each experiment. ANOVA was utilized for above comparisons.

Next we explored ATP linked respiration (basal respiration minus oligomycin sensitive) in platelets in our porcine model at baseline and at the end of the observation period (**Fig 7A**). Platelets were investigated because the potential to have repeated measurements in a representative tissue over time that would not require multiple biopsies or invasive procedures, the possibility to translate changes seen in these preclinical models to future measurements in patients, as well as the clinical relevancy given the importance of platelets in coagulopathy and inflammation. HS/R led to a significant decrease in ATP-linked respiration, while CO treated pigs appeared similar to sham. Additionally CO increased mitochondrial reserve capacity (which is measured as FCCP stimulated maximal respiration minus basal respiration, and represents the maximal respiratory capacity of the mitochondria; **Fig 7B**). These platelet mitochondrial changes were concomitant with CO-induced attenuation of HS/R-induced platelet activation as measured by increased cell surface expression of CD62p by flow cytometry (HS/R showed a 2.33±0.1 fold increase over sham pigs at a 2 hour time point following resuscitation, *p<0.05; vs. 1.64±0.08 fold increase over sham pigs; P<0.05 compared to untreated shock and resuscitation; **Fig 7C**).

**Fig 8:**
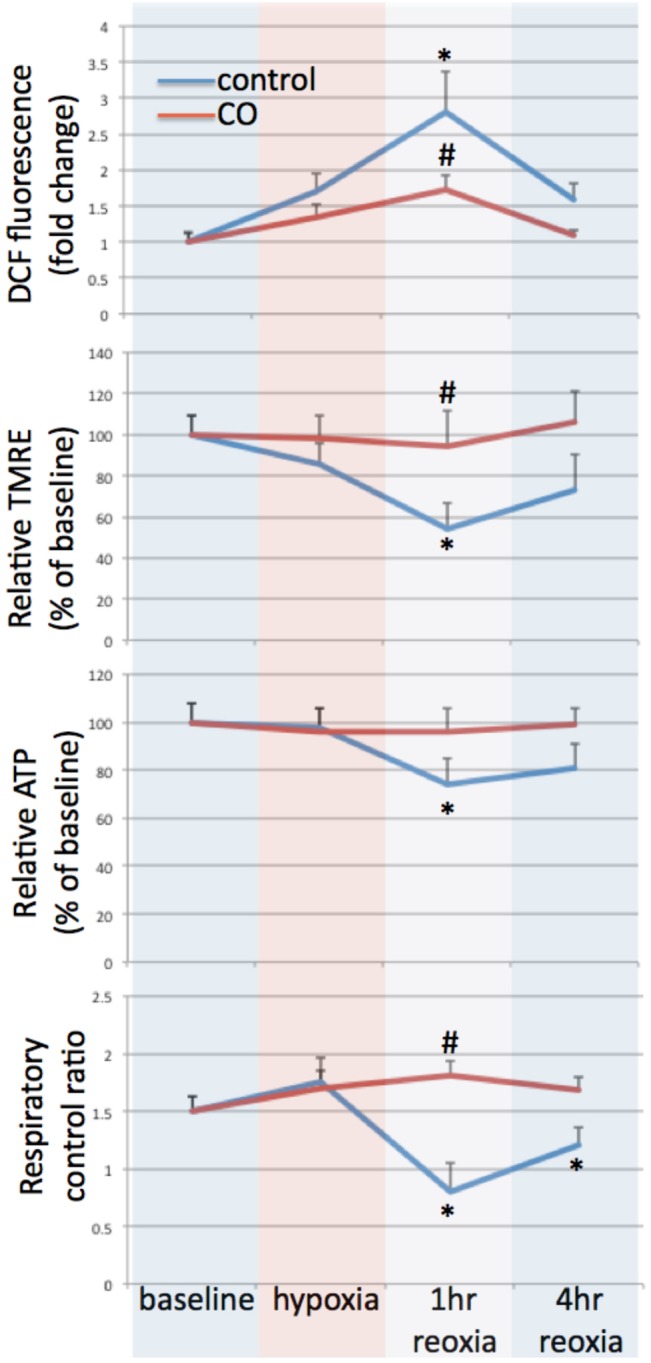
CO limits hypoxia-induced oxidant and mitochondrial injury in murine skeletal muscle ex-vivo. Murine skeletal muscle was freshly collected from mice after perfusion with cold PBS, cut into strips (2 X 2 X 10mm) and exposed ex vivo to hypoxia (1% O_2_) for one hour followed by reoxia for up to 4 hours. Some samples were treated with CO (250 ppm) during hypoxia. Measurements of ROS by DCF fluorescence, relative mitochondrial membrane potential by TMRE fluorescence, relative ATP levels, and respiratory control ratio (RCR) were determined at baseline, end of hypoxia, 1 and 4 hours. Hypoxia induced increased DCF fluorescence and a drop in mitochondrial membrane potential; however statistically significant differences in these parameters, as well as reductions in ATP and RCR were not seen until after reoxygenation (*P<0.05 compared to baseline). All parameters had returned to or were trending back to baseline by 4 hours after reoxia. CO treatment limited these hypoxia/reoxia-induced changes (#P<0.05 compared to hypoxia+1 hour reoxia). Each experiment was performed in duplicate and repeated three times. T-tests were used for the above comparisons.

The influence of CO on mitochondrial injury and bioenergetics was further investigated in murine striated muscle in hypoxia *ex vivo*. Freshly harvested murine skeletal muscle was exposed to hypoxia (1% oxygen) for one hour and then re-oxygenated for up to 4 hours. Some samples were treated with CO (250 ppm) during the hypoxic period. At the end of the hypoxic period there was a trend towards increasing reactive oxygen species formation and mitochondrial depolarization, but there was no change in RCR or ATP levels (**Fig 8**). However, after 1 hour of reoxygenation, there were significant changes in mitochondrial injury parameters and bioenergetics, including increased DCF fluorescence as a marker of mitochondrial injury (2.8±0.56 fold increase from baseline; P<0.05), decreased TMRE positive staining suggesting mitochondrial depolarization (54±18% of baseline; P<0.05), decreased RCR and decreased ATP levels (53±17% and 74±11% of baseline, respectively; P<0.05). At a four-hour time point following reoxygenation these parameters were improving, suggesting compensatory mechanisms of recovery. CO treatment protected against these hypoxia/reoxia–induced changes.

## Discussion

Inhaled CO as adjunct to standard resuscitation ameliorated the signs of shock, delayed circulatory collapse, and showed a trend towards improved survival in multiple animal models of HS/R. CO was also associated with a dose dependent reduction in organ injury and inflammation, with no detrimental effect on systemic hemodynamic or oxygenation parameters, and with safe increments in carboxyhemoglobin. Furthermore, CO protected against HS/R-induced oxidant injury and loss of mitochondrial function.

Others and we have demonstrated CO to be protective in murine models of hemorrhagic shock, ischemia/reperfusion, and sepsis[10, 21–23]. We now demonstrate that inhaled CO delivered into the hypotensive period prior to resuscitation can be safely administered in a pre-clinical model of hemorrhagic shock, and that it can effectively and dose-dependently limit organ injury and inflammation. These findings have also an important translational component, as this could represent a simple, safe and effective intervention that could eventually be administered by first responders in the field, with the potential to positively impact outcome in trauma victims. Although the findings hereby presented represent strong pre-clinical data, it is important to underscore that this study was not powered for the assessment of mortality, and thus further studies may be warranted to evaluate this further. Additionally, a recognized shortcoming of this study was the resuscitation fluid choice and volume in the porcine study does not represent the modern trauma care, which focuses on blood product resuscitation and smaller volumes. However, this is a representative model and it is unlikely that these limitations in the model will restrict potential translatability into future clinical trials.

The mechanisms by which CO exerts protection are likely multifactorial. However, previous work, coupled with these data suggest specific processes. Our group previously demonstrated in murine hemorrhagic shock that CO paradoxically prevented tissue hypoxia[10]. This could be explained by several possible mechanisms. One is that CO may protect microvascular function through amelioration of the inflammatory response and perhaps limitation of endothelial injury and activation[4, 24, 25]. As microvascular function is protected, regional O_2_ delivery is preserved and thus, O_2_ availability within the cell is maintained. Alternatively, it has been long been known, mainly from the experience with CO intoxicated victims, that CO can impair mitochondrial respiration and profoundly decrease O_2_ utilization. We now provide data to support the notion that low dose CO may help mitochondria down regulate O_2_ consumption, preventing cellular hypoxia. Importantly, we have previously shown that HO-1 over-expression can limit oxygen consumption in hypoxia[7], and now demonstrate that CO can limit oxygen consumption and can increase intracellular oxygen levels in hypoxic hepatocytes, suggesting that this HO-1 effect can be mimicked by administration of exogenous CO. CO-mediated decreases in oxygen consumption may be a protective mechanism to explain why CO treatment limited the development of shock (higher pH, and lower base deficit and lactate levels) following hemorrhage.

Influences of CO on mitochondrial respiration may also have profound effects to minimize subsequent reperfusion injury[26–30]. The findings in this manuscript demonstrate that CO treatment in mice and pigs limited hemorrhage and reperfusion induced mitochondrial injury in muscle and platelets. Most markers of mitochondrial injury were not increased until after reperfusion. The effect of hemorrhage on mitochondrial function was relatively negligible during the shock period or immediately following resuscitation. These *in vivo* data suggest that CO limits mitochondrial dysfunction and protects against the uncoupling effects of reperfusion injury. Furthermore, *ex vivo* studies illustrate that there is less hypoxia/reoxia-induced oxidant stress, mitochondrial injury, and decrease in ATP levels with CO exposure. Cumulatively, these data suggest that most injury occurs with reperfusion or reoxygenation.

Because CO influences oxygen consumption rates and may decrease the level of intracellular hypoxia, there may be less mitochondrial injury during hypoxia, which manifests as oxidant stress and bioenergetics failure most notably after reoxygenation. Moreover, the presence of CO bound to heme proteins at the time of reoxygenation or reperfusion may also blunt the influence of reintroduction of oxygen at the level of injured mitochondrion and to other pro-oxidant enzymes in other subcellular compartments [31]. Essentially creating a situation of slower reintroduction of oxygen at the cellular level to limit potentially injurious oxidant stress producing enzymatic reactions. Other mechanisms that could potentially explain the protective effects of CO on mitochondrial function which were not explored in this manuscript include its known ability to promote mitochondrial quality control processes such as fission, fusion, mitophagy and biogenesis[32].

Carbon monoxide is known to possess potential vasodilatory properties, presumably through activation of guanylate cyclase[33]. However, we did not find any statistical or clinically significant differences in any of the macro-hemodynamic parameters, systemic oxygenation indices that we monitored or even in the amount of blood shed during the shock period. This is important because we now provide evidence that this therapeutic approach is safe in a valid, reproducible pre-clinical model, and thus raise the translational value of the present findings. Moreover, in the porcine model the carboxyhemoglobin peaked around 4.5% after 30 minutes of inhaling 250 ppm in and FiO_2_ of 40%. This is a level that is safe and not associated with any known toxicity.

In conclusion, CO may prove to be a simple, safe and efficient resuscitative adjunct in the treatment of trauma and hemorrhagic shock, with a potential to impact patient centered outcomes. These data also suggest that CO may confer protection by prevention bioenergetic and mitochondrial failure, by limiting inflammation and oxidative stress and potentially, and provides indirect evidence of a possible impact on microvascular flow. Although CO carries the stigma of toxicity, the medical use of CO holds great promise. Further investigations are warranted to further reveal the true potential of this adjunct therapy in the management of victims of traumatic hemorrhagic shock.

## Supporting Information

S1 Fig Schema of murine models of hemorrhagic shock and resuscitation. Moderate shock was performed by a controlled hemorrhage to a MAP of 25 mmHg. This was maintained for a total of 120 minutes. Mice received air or carbon monoxide for 30 minutes starting 90 minutes into the hypotensive period. Mice were resuscitated with lactated Ringers (LR) at two times the maximum volume of shed blood and were euthanized 4 hours after resuscitation. For the severe shock model, mice were hemorrhage to a MAP of 20 mmHg. Air or CO (250 ppm) was initiated after this MAP was achieved and was continued for 30 minutes. Mice were either euthanized and arterial blood gases were measured or then allowed to stay at this pressure until the compensation endpoint was reached. After which they were euthanized. The severe shock-survival model mice were hemorrhaged to a MAP of 20 mmHg and then maintained at this pressure for 30 minutes. They were then resuscitated with their own shed blood to reach a MAP of 25 mmHg, and were then maintained at this pressure for 60 more minutes. Air or CO therapy (250 ppm for 30 minutes) was initiated 30 minutes into this period with a maintained MAP of 25 mmHg. Mice were then resuscitated with LR at two times the maximum volume of shed blood and were observed for 36 hours. Mice were monitored during these experiments continuously during the surgical procedure then checked upon at least every two hours post-operatively. Thew were monitored for mobility, and signs of distress. Buprenex was administered every 6 hours for pain at the dose of 0.10 mg/kg. If a mouse appeared in distress or pre-morbid they were euthanized with CO_2_ asphyxiation.
(TIFF)

S2 Fig Schema for porcine model of hemorrhagic shock and resuscitation. A 30 minute stabilization period was allowed after the initial surgical interventions for arterial and venous catheter placements. Following this pigs were bled at a rate of 20 mL/minute to achieve a MAP of 30 mmHg. Once obtained they were maintained at this MAP for 90 minutes. If th MAP increased above 40 mmHg, they were bled at a rate of 60 mL/minute back to a MAP of 30 mmHg. Randomization to control or CO (250 ppm for 30 mnutes). Pigs were initially resuscitated with Hextend at a 1:1 volume of total shed blood. Subsequent resuscitation was with LR. Pigs were euthanized at 240 minutes after the initial resuscitaion.
(TIFF)

S3 Fig Porcine hemorrhagic shock survival over the course of the experiment. Time “0 minutes” represents the start of the hypotensive period. Survival was monitored until 4 hours after resuscitation. Mortality rate was 25% in the control group (n = 4 of 16) and 8.3% in the CO treated group (n = 1 of 12).
(JPG)

S1 Table Total blood volume/hemorrhage volume from murine shock experiments. Murine shock models include “Moderate,” “Severe Acute,” and “Severe Survival.” In the “moderate shock’ model mice were hemorrhaged to a MAP of 25 mmHg and maintained at this MAP for 120 minutes. CO or control therapy was initiated 90 minutes into establishing a MAP of 25 mmHg. This model was used for measurements of organ injury (ALT, Myeloperoxidase activity, and lipid peroxidation), as well as systemic inflammation by serum cytokines. In “severe acute” mice were bled to a MAP of 20 mmHg and maintained at this pressure for 30 minutes or until mice could no longer compensate. No blood was returned in this model. In “severe survival” mice were hemorrhaged to a MAP of 20 mmHg and maintained at this pressure for 30 minutes. Mice were then resuscitated with shed blood to a MAP of 25 and maintained at this pressure for an additional 60 minutes. CO or control therapy was initiated 30 minutes into re-establishing a MAP of 25 mmHg. See methods for further details. Estimated total blood volume was calculated assuming a murine blood volume of 78 microliters per gram.
(DOCX)

S2 Table Total blood volume/hemorrhage volume from porcine shock experiments. Sham pigs underwent anesthesia and surgical manipulation without hemorrhage. Randomization of shocked pigs to control or CO therapy was carried out 55 minutes into the hypotensive period. CO therapy was started 60 minutes after the establishment of a MAP of 30 mmHg.
(DOCX)
