# Supplementary figures and images for: Inhaled Carbon Monoxide Protects against the Development of Shock and Mitochondrial Injury following Hemorrhage and Resuscitation
Source: PLoS One. 2015 Sep 14;10(9):e0135032. doi: 10.1371/journal.pone.0135032 (PMC4569171; doi:10.1371/journal.pone.0135032)

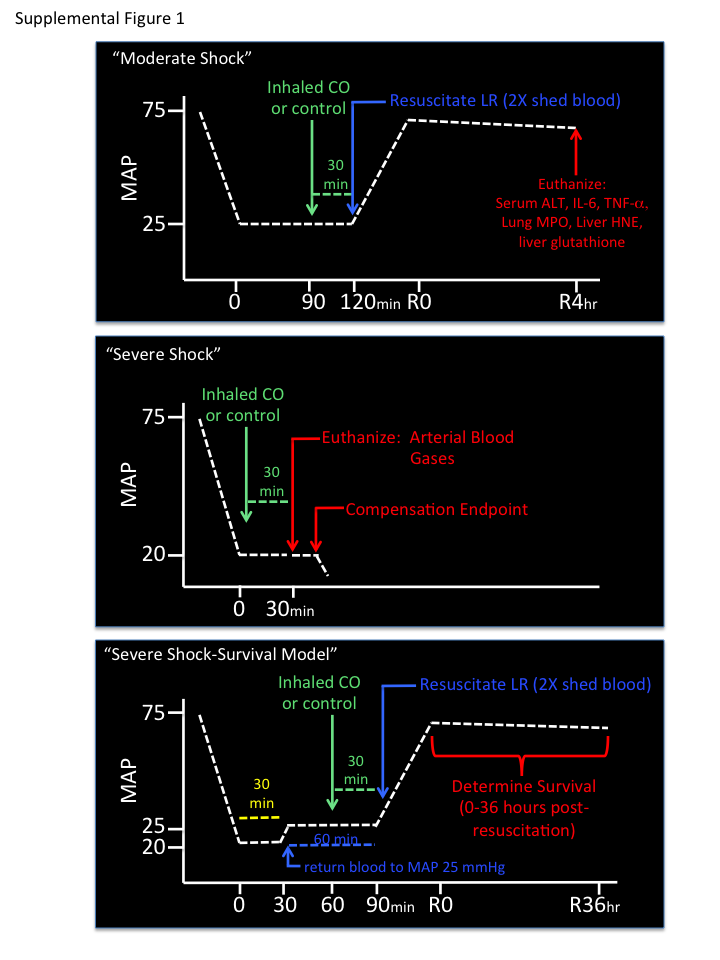

Supplement: S1 Fig — Moderate shock was performed by a controlled hemorrhage to a MAP of 25 mmHg. This was maintained for a total of 120 minutes. Mice received air or carbon monoxide for 30 minutes starting 90 minutes into the hypotensive period. Mice were resuscitated with lactated Ringers (LR) at two times the maximum volume of shed blood and were euthanized 4 hours after resuscitation. For the severe shock model, mice were hemorrhage to a MAP of 20 mmHg. Air or CO (250 ppm) was initiated after this MAP was achieved and was continued for 30 minutes. Mice were either euthanized and arterial blood gases were measured or then allowed to stay at this pressure until the compensation endpoint was reached. After which they were euthanized. The severe shock-survival model mice were hemorrhaged to a MAP of 20 mmHg and then maintained at this pressure for 30 minutes. They were then resuscitated with their own shed blood to reach a MAP of 25 mmHg, and were then maintained at this pressure for 60 more minutes. Air or CO therapy (250 ppm for 30 minutes) was initiated 30 minutes into this period with a maintained MAP of 25 mmHg. Mice were then resuscitated with LR at two times the maximum volume of shed blood and were observed for 36 hours. Mice were monitored during these experiments continuously during the surgical procedure then checked upon at least every two hours post-operatively. Thew were monitored for mobility, and signs of distress. Buprenex was administered every 6 hours for pain at the dose of 0.10 mg/kg. If a mouse appeared in distress or pre-morbid they were euthanized with CO2 asphyxiation. (TIFF) [file pone.0135032.s001.tiff]

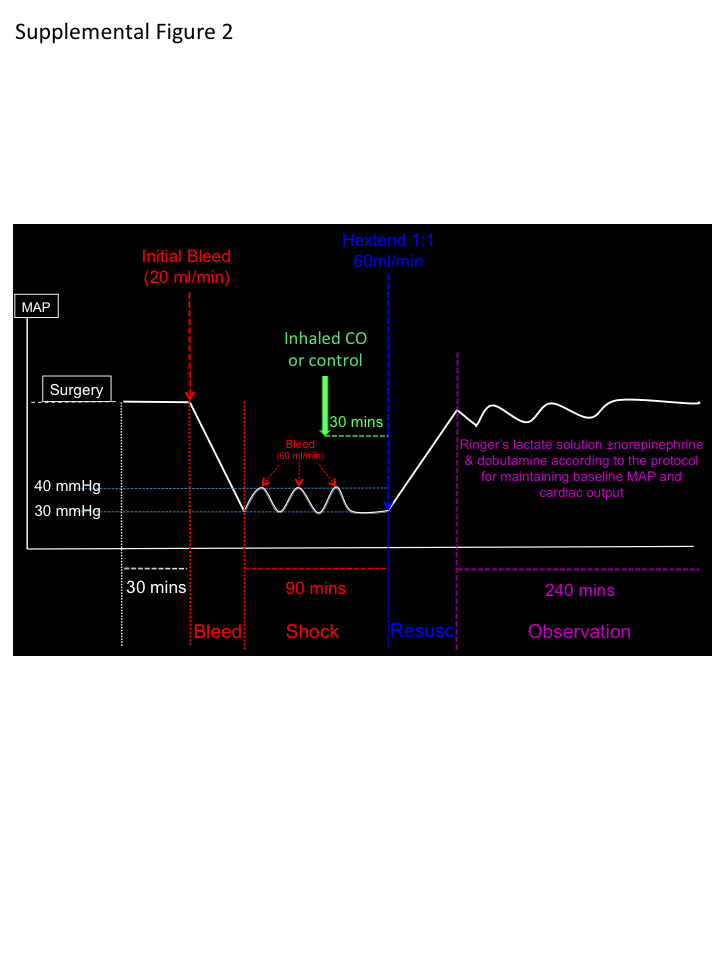

Supplement: S2 Fig — A 30 minute stabilization period was allowed after the initial surgical interventions for arterial and venous catheter placements. Following this pigs were bled at a rate of 20 mL/minute to achieve a MAP of 30 mmHg. Once obtained they were maintained at this MAP for 90 minutes. If th MAP increased above 40 mmHg, they were bled at a rate of 60 mL/minute back to a MAP of 30 mmHg. Randomization to control or CO (250 ppm for 30 mnutes). Pigs were initially resuscitated with Hextend at a 1:1 volume of total shed blood. Subsequent resuscitation was with LR. Pigs were euthanized at 240 minutes after the initial resuscitaion. (TIFF) [file pone.0135032.s002.tiff]

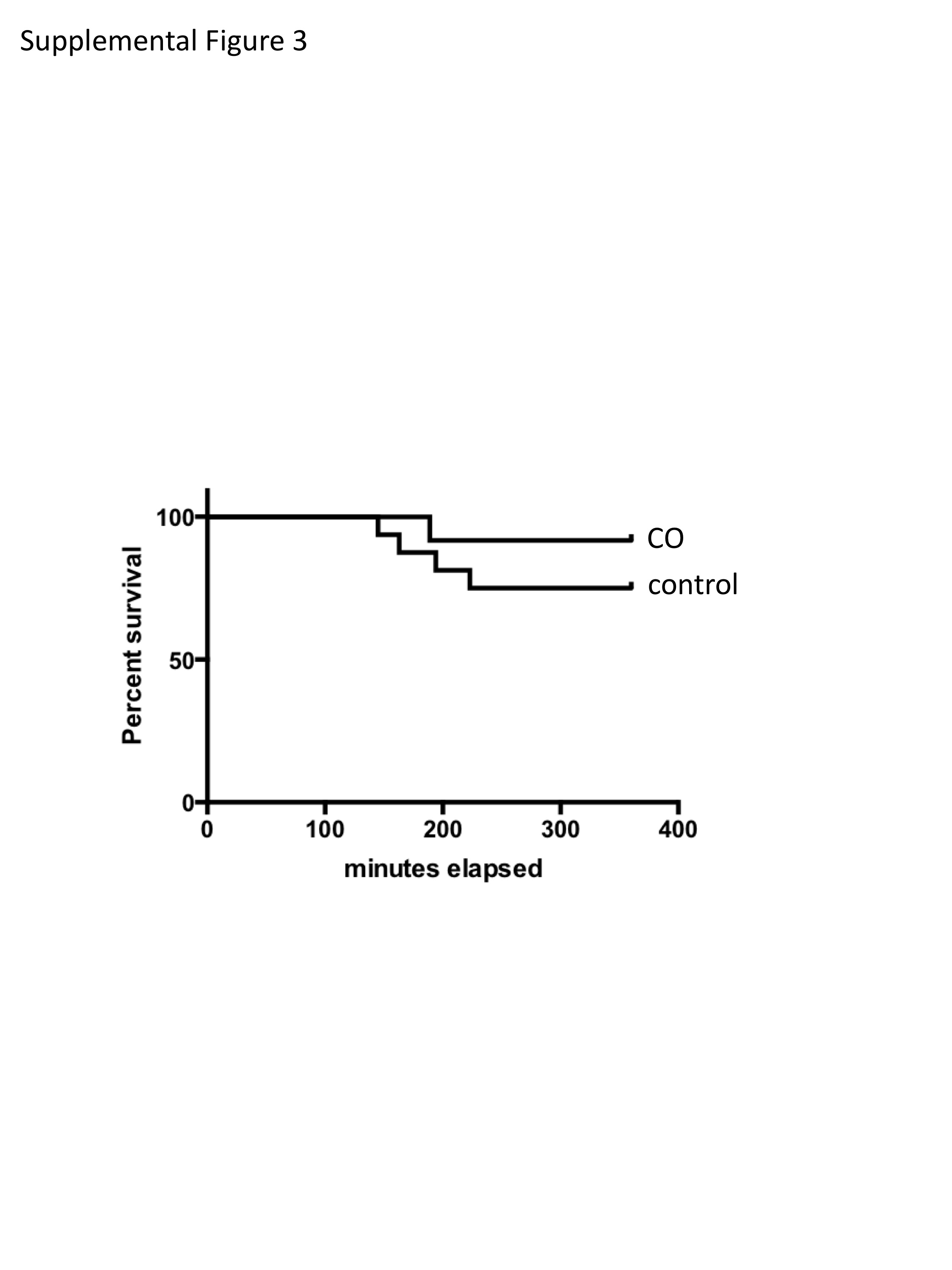

Supplement: S3 Fig — Time “0 minutes” represents the start of the hypotensive period. Survival was monitored until 4 hours after resuscitation. Mortality rate was 25% in the control group (n = 4 of 16) and 8.3% in the CO treated group (n = 1 of 12). (JPG) [file pone.0135032.s003.jpg]
