# Supplementary material for: Inhaled Carbon Monoxide Protects against the Development of Shock and Mitochondrial Injury following Hemorrhage and Resuscitation
Source: PLoS One. 2015 Sep 14;10(9):e0135032. doi: 10.1371/journal.pone.0135032 (PMC4569171; doi:10.1371/journal.pone.0135032)
Supplement: S1 Table — Murine shock models include “Moderate,” “Severe Acute,” and “Severe Survival.” In the “moderate shock’ model mice were hemorrhaged to a MAP of 25 mmHg and maintained at this MAP for 120 minutes. CO or control therapy was initiated 90 minutes into establishing a MAP of 25 mmHg. This model was used for measurements of organ injury (ALT, Myeloperoxidase activity, and lipid peroxidation), as well as systemic inflammation by serum cytokines. In “severe acute” mice were bled to a MAP of 20 mmHg and maintained at this pressure for 30 minutes or until mice could no longer compensate. No blood was returned in this model. In “severe survival” mice were hemorrhaged to a MAP of 20 mmHg and maintained at this pressure for 30 minutes. Mice were then resuscitated with shed blood to a MAP of 25 and maintained at this pressure for an additional 60 minutes. CO or control therapy was initiated 30 minutes into re-establishing a MAP of 25 mmHg. See methods for further details. Estimated total blood volume was calculated assuming a murine blood volume of 78 microliters per gram. (DOCX) [file pone.0135032.s004.docx]

| **Table S1**. Total blood volume/hemorrhage volume from murine shock experiments. | | | | |
| --- | --- | --- | --- | --- |
| Model/group | grams | Estimated total blood volume (μL) | Maximum shed blood volume (μL) | Maximum returned blood volume (μL) |
| *Moderate* |  |  |  |  |
| Sham (n=8) | 25.1±0.9 | 1958±70 | n/a | n/a |
| Sham + CO (n=8) | 25.0±0.8 | 1950±62 | n/a | n/a |
| Shock (n=8) | 25.6±1.1 | 1997±86 | 833±121 | 84±31 |
| Shock + CO (n=8) | 25.3±1.2 | 1973±94 | 814±136 | 72±48 |
| *Severe Acute* |  |  |  |  |
| Sham (n=18) | 24.8±1.3 | 1934±101 | n/a | n/a |
| Sham + CO (n=18) | 25.1±1.0 | 1958±78 | n/a | n/a |
| Shock (n=18) | 24.6±1.2 | 1919±94 | 1001±119 | n/a |
| Shock + CO (n=18) | 24.9±0.9 | 1942±70 | 1031±131 | n/a |
| *Severe Survival* |  |  |  |  |
| Sham (n=20) | 25.1±0.7 | 1958±55 | n/a | n/a |
| Sham + CO (n=20) | 25.6±0.9 | 1997±70 | n/a | n/a |
| Shock (n=20) | 25.2±0.8 | 1966±62 | 1088±104 | 311±92 |
| Shock + CO (n=20) | 25.0±0.7 | 1950±55 | 1017±125 | 286±108 |
| Murine shock experiments include “Moderate, “ “Severe Acute,” and “Severe Survival.” In the “moderate shock’ model mice were hemorrhaged to a MAP of 25 mmHg and maintained at this MAP for 120 minutes. CO or control therapy was initiated 90 minutes into establishing a MAP of 25 mmHg. This model was used for measurements of organ injury (ALT, Myeloperoxidase activity, and lipid peroxidation), as well as systemic inflammation by serum cytokines. In “severe acute” mice were bled to a MAP of 20 mmHg and maintained at this pressure for 30 minutes or until mice could no longer compensate. No blood was returned in this model. In “severe survival” mice were hemorrhaged to a MAP of 20 mmHg and maintained at this pressure for 30 minutes. Mice were then resuscitated with shed blood to a MAP of 25 and maintained at this pressure for an additional 60 minutes. CO or control therapy was initiated 30 minutes into re-establishing a MAP of 25 mmHg. See methods for further details. Estimated total blood volume was calculated assuming a murine blood volume of 78 microliters per gram. | | | | |

**Table S1**. Total blood volume/hemorrhage volume from murine shock experiments. Murine shock models include “Moderate, “ “Severe Acute,” and “Severe Survival.” In the “moderate shock’ model mice were hemorrhaged to a MAP of 25 mmHg and maintained at this MAP for 120 minutes. CO or control therapy was initiated 90 minutes into establishing a MAP of 25 mmHg. This model was used for measurements of organ injury (ALT, Myeloperoxidase activity, and lipid peroxidation), as well as systemic inflammation by serum cytokines. In “severe acute” mice were bled to a MAP of 20 mmHg and maintained at this pressure for 30 minutes or until mice could no longer compensate. No blood was returned in this model. In “severe survival” mice were hemorrhaged to a MAP of 20 mmHg and maintained at this pressure for 30 minutes. Mice were then resuscitated with shed blood to a MAP of 25 and maintained at this pressure for an additional 60 minutes. CO or control therapy was initiated 30 minutes into re-establishing a MAP of 25 mmHg. See methods for further details. Estimated total blood volume was calculated assuming a murine blood volume of 78 microliters per gram.
