# Supplementary material for: Inhaled Carbon Monoxide Protects against the Development of Shock and Mitochondrial Injury following Hemorrhage and Resuscitation
Source: PLoS One. 2015 Sep 14;10(9):e0135032. doi: 10.1371/journal.pone.0135032 (PMC4569171; doi:10.1371/journal.pone.0135032)
Supplement: S2 Table — Sham pigs underwent anesthesia and surgical manipulation without hemorrhage. Randomization of shocked pigs to control or CO therapy was carried out 55 minutes into the hypotensive period. CO therapy was started 60 minutes after the establishment of a MAP of 30 mmHg. (DOCX) [file pone.0135032.s005.docx]

| **Table S2**. Total blood volume/hemorrhage volume from porcine shock experiments. | | | | |
| --- | --- | --- | --- | --- |
|  | Kg | Total blood volume (mL; calculated) | Hemorrhaged volume (mL) | % of Total blood volume |
| Sham (n=7) | 32.6±2.8 | 2197±192 | N/A | N/A |
| Shock (n=15) | 31.4±2.9 | 2184±360 | 1051±352 | 51.8±11.9 |
| Shock + CO (n=12) | 30.9±4.1 | 2077±277 | 946±279 | 45.5±13.4 |
| Sham pigs underwent anesthesia and surgical manipulation without hemorrhage. Randomization of shocked pigs to control or CO therapy was carried out 55 minutes into the hypotensive period. CO therapy was started 60 minutes after the establishment of a MAP of 30 mmHg. There are no statistically significant differences between groups. | | | | |

**Table S2**. Total blood volume/hemorrhage volume from porcine shock experiments. Sham pigs underwent anesthesia and surgical manipulation without hemorrhage. Randomization of shocked pigs to control or CO therapy was carried out 55 minutes into the hypotensive period. CO therapy was started 60 minutes after the establishment of a MAP of 30 mmHg.
